# Dietary RNAs: New Stories Regarding Oral Delivery

**DOI:** 10.3390/nu7053184

**Published:** 2015-04-30

**Authors:** Jian Yang, Kendal D. Hirschi, Lisa M. Farmer

**Affiliations:** 1 United States Department of Agriculture/Agriculture Research Service, Children’s Nutrition Research Center, Department of Pediatrics, Baylor College of Medicine, Houston, TX 77030, USA; E-Mails: kendah@bcm.edu (K.D.H.); lmfarmer@bcm.edu (L.M.F.); 2 Vegetable and Fruit Improvement Center, Texas A&M University, College Station, TX 77845, USA

**Keywords:** dietary microRNAs, Next Generation Sequencing (NGS), microvesicle, serum

## Abstract

microRNAs (miRNAs), a class of small RNAs, are important regulators of various developmental processes in both plants and animals. Several years ago, a report showed the detection of diet-derived plant miRNAs in mammalian tissues and their regulation of mammalian genes, challenging the traditional functions of plant miRNAs. Subsequently, multiple efforts have attempted to replicate these findings, with the results arguing against the uptake of plant dietary miRNAs in healthy consumers. Moreover, several reports suggest the potential for “false positive” detection of plant miRNAs in human tissues. Meanwhile, some research continues to suggest both the presence and function of dietary miRNAs in mammalian tissues. Here we review the recent literature and discuss the strengths and weaknesses of emerging work that suggests the feasibility of dietary delivery of miRNAs. We also discuss future experimental approaches to address this controversial topic.

## 1. Introduction

The majority of people in the world live primarily on plant-based diets. Like the medley of bioactive compounds found in plants, our diet also contains different small RNAs, including microRNAs (miRNAs) that are 19–24 nucleotides in length [1]. In planta, these small RNAs help regulate metabolism, growth and stress responses by attaching to specific mRNAs and modifying their translation. Could plant miRNAs be regulating gene expression within the consumer?

In animal cells, miRNAs inhibit translation or stability of mRNA transcripts by binding to them with largely imperfect complementarity [1]. By recognition of hundreds of mRNA transcripts, a single miRNA can shift the entire transcription profile of a cell, consistent with a pivotal role for miRNAs in establishing and maintaining tissue identity [2]. In contrast, plant miRNAs base pair to their targets with essentially perfect complementarity and effect RNA cleavage and degradation rather than translational repression [1]. Regardless of mechanism, an organism's endogenous miRNA system is used to regulate many processes including growth and adaptive responses. Besides functioning locally in animal cells, a model has been suggested whereby miRNAs are encapsulated by microvesicles (MVs), which are shed from almost all cell types and have the potential to travel to and interact with specific target cells [3,4]. In fact, some studies have shown that stable miRNAs in mammalian blood may serve as a class of biomarkers [5].

Delivery of a miRNA-like species called short interfering RNA (siRNA), differing from miRNA in that siRNAs are perfectly complementary to their targets and exhibit plant miRNA-like transcript cleavage and degradation, underlies RNA interference (RNAi) approaches to alter animal gene expression in experimental biology. The transparent nematode *Caenorhabditis elegans* is a great model for demonstrating RNAi, because siRNA delivery is relatively simple yet extremely effective. A membrane transporter, SID1, participates in this RNA delivery [6], which appears to be microvesicle independent. Bacteria engineered to express a desired siRNA can be fed to the worms and will transfer their consignment to the worm via the intestinal tract [7]. This SID1-dependent “delivery by feeding” is efficient and economical; however, a definitive role for SID1 homologs in higher eukaryotes has not been established.

A striking report suggests that ingested plant miRNAs are transferred to blood, accumulate in tissues, and exert regulation of transcripts in consuming animals [8]. However, evidence now suggests that the claims of delivery and effect [8] may not be generally applicable to all consumers [9,10]. Meanwhile, other reports show evidence of dietary plant miRNA uptake and function in relation to certain miRNAs/consumers [11,12,13,14]. Here we seek to review the current state of knowledge, suggest future directions, and consider how these findings may impact nutrition, medicine and agrobiotechnology.

## 2. Overview of Main Techniques Used to Measure microRNA Levels

Determining the identity and expression levels of miRNAs is of paramount importance in these dietary studies. Traditional methods for measuring miRNAs in a total RNA sample include microarrays and quantitative Reverse Transcription-Polymerase Chain Reaction (qRT-PCR) methodologies. Microarray analysis will not work to measure plant-based miRNAs in consumers simply because the plant-based miRNAs are not included on the available animal hybridization chips. There are also some complications with qRT-PCR. For some RT-PCR applications a ligation step is used. However, plant miRNAs contain a unique 2’-O-methylation on the ribose of the 3’ nucleotide that is not present in the animal miRNAs and is shown to negatively affect the ligation step in qRT-PCR [15]. This difference may lead to underrepresentation of plant sequences detected from mixed plant and animal libraries [10,16,17]. Next Generation Sequencing (NGS) and digital PCR (dPCR) are newer technologies that have also been implemented. NGS offers many advantages over other methods of profiling miRNAs, such as more efficient sample throughput and the capability to unambiguously identify miRNAs [18]. The strategy for dPCR requires that a sample be diluted and divided into millions of separate reaction cells so that each contains one or no copies of the sequence of interest [19]. This technology directly counts the number of target molecules, whereas conventional qPCR relies on reference standards or endogenous controls.

Regardless of the technique used, too much sensitivity rather than too little is often a problem. The pervasiveness of nucleic acid contamination can results in false positives that confound experimental studies [20,21]. We will not belabor the technical shortcomings used in various studies; however, the reader should keep in mind that multiple approaches should be used and that regardless of methodology, when low levels of a miRNA are detected, the first consideration of both the investigator and the reader should be contamination or background signal, not exclusively low level expression.

## 3. Initial Publication on Uptake and Function of Dietary microRNAs in Mammals

In a groundbreaking publication out of Nanjing University in China, diet-based plant miRNAs were found in human blood and animal tissue samples [8]. We will refer to this work throughout this review as the “Zhang group study”. In this work, approximately 30 distinguishable plant miRNAs were identified by Solexa sequencing (a type of NGS) in a sampling of pooled whole sera from men and women (10 pools with each pool including approximately 10 individual’s sera) whose diets were predominately comprised of plant foods (vegetables, fruit, rice, *etc.*) [8]. Approximately 10 of these miRNAs had modest expression levels in the sera (reads > 300). The presence of 2′-O-methylation on the miRNAs was used to confirm that they were diet-derived. These results were not confined to the Chinese population used in this study, as the authors documented that other exogenous dietary plant miRNAs could be found circulating in livestock. In human serum samples, two rice miRNAs named MIR156a and MIR168a were present in particularly high concentrations. To establish that MIR168a is delivered through the diet, the authors performed animal feeding studies in which they compared serum miRNA profiles among mice fed chow, a rice-based diet, or chow supplemented with exogenous synthetic MIR168a. The MIR168a was found only in its mature form and at robust levels in the tissues from the rice-fed and MIR168a-supplemented chow-fed animals. Additional experiments suggested that these plant miRNAs could endure conditions that mimic the acidic environment of the stomach. Taken together, these observations suggest that dietary plant miRNAs survive ingestion and digestion, transverse the gut lining and end up in circulation.

Plant-derived miRNAs appear to be delivered during ingestion, but are they active? Using a bioinformatics approach, the investigators deduced that MIR168a had the capacity to bind to a mRNA that is translated into a low-density lipoprotein receptor adapter protein 1 (LDLRAP1), which controls blood cholesterol levels. If LDLRAP1 mRNA levels drop, cholesterol in the blood goes up. To test this *in vivo*, mice were fed a control diet, a rice-based diet, or a MIR168a-enriched diet. In animals given the rice and MIR168a-enriched diets, the authors found that liver and blood levels of MIR168a increased, while LDLRAP1 protein levels decreased and their cholesterol levels subsequently increased. Conversely, a compound that specifically inhibited MIR168a from binding to the LDLRAP1 mRNA blocked these effects. These findings are consistent with cross-kingdom regulation by dietary miRNAs. To summarize, the Zhang group demonstrated both delivery and biological effects of dietary miRNAs in animal tissues.

## 4. Contradicting Evidence for Dietary Uptake of microRNA

Numerous details of the Zhang group study have been appraised [9,22]. For example, less than a handful of plant miRNAs are regularly detected in humans; however, there is variability among samples. Levels of two of the dietary miRNAs in this publication were found at approximately the same levels of some endogenous miRNAs. If this is true, why have these specific miRNAs been virtually undetectable in the majority of other studies looking at circulating miRNA levels? Furthermore, there were only minimal changes in dietary miRNA levels following feeding.

Of the 80 public datasets analyzed for evidence of plant miRNAs in animal fluids and tissues [23], approximately 50 yielded reads from fewer than three apparent plant miRNA species. The plant-based miRNAs were present with median counts of less than six per million animal miRNA reads (range: 0.4–1089). MIR168a was registered somewhat more often, at a median of 181 counts per million animal miRNA reads. In several reviewed datasets, a variant of MIR168a was detected in animals that did not receive food containing that miRNA [23]. To approximate the dietary exposure of mice in this study [8] a 55 kg human would have to eat approximately 33 kg of cooked rice per day [24]. These findings have led several groups to the conclusion that MIR168a reads in vertebrate samples are not biologically relevant.

### 4.1. Unsuccessful Replication of the Seminal Study Using Rice Diet in Mice

A comprehensive effort by Monsanto and miRagen researchers failed to replicate the Zhang group’s work [10]. In this replication study, mice were given three dietary formulations: standard chow, a diet nutritively equivalent to chow but supplemented with 41% rice, or a raw rice diet. Using these different feeding regimes, the authors were unable to demonstrate consistent dietary delivery of plant-based miRNAs to the animals. In fact, little or no plant miRNA was found in the blood or organs of mice fed with the described diets. As such, they were unable to demonstrate that specific dietary miRNAs had any impact on gene regulation in the consumers.

### 4.2. Human Athletes, Honeybees Did Not Show Obvious Absorption of miRNAs from Plant Diets

Multiple other independent studies have reported negative results upon feeding miRNA-rich plant-based diets to a variety of insects and animals. In all attempts, plant miRNAs were detected at substantial levels in the diets, but were undetectable in animal fluids and tissues. Snow *et al.* selected three highly conserved, easily detectable plant miRNA species (MIR156a, MIR159a, and MIR169a) that have been demonstrated as abundant in fruits that are commonly found in Western diets (e.g., apples, bananas, avocados). Examination of plasma from healthy athletes that were documented to routinely ingest fruits containing high levels of MIR156a, MIR159a, and MIR169a revealed that while they could detect endogenous human miRNA miR-16 at high levels, the plasma samples were devoid of diet-derived plant miRNAs. This suggests that plant miRNAs are not maintained circulating at a steady-state levels in human blood. The authors performed a similar analysis on honeybees, the primary food sources of which are honey, nectar, and pollen, all of which contain appreciable levels of MIR156a, MIR159a, and MIR169a. Gut tissue isolated from their abdomens contained appreciable levels of the endogenous miRNA let-7, while the plant miRNAs of interest were either wholly undetectable (MIR159a and MIR169a), or found at negligible levels of less than 1 copy per cell (MIR156a), thus reflecting the findings in human consumers [16].

### 4.3. Neither Normal Mice nor miR-21 Null Mice Showed Any Absorption of Dietary miRNAs

In a third approach to test the efficacy of diet-induced plant miRNA uptake, Snow *et al.* fed mice with vegetarian or soy-enriched diets, both of which are replete in MIR156a, MIR159a, and MIR169a, or a casein- and lard-based diet that is devoid of the aforementioned plant miRNAs but contains endogenous animal miRNAs. Animals consumed comparable volumes of each diet and their plasma were subsequently assayed for the presence of plant miRNAs. While miR-16 was abundant, an exceedingly low level (<1 copy per cell) of MIR156a was detected in organ tissues, while MIR159a and MIR169a were undetectable in any tissues. The concern that perhaps feeding mice processed diets may have affected the efficiency of miRNA absorption after ingestion led the authors to feed a population of mice unprocessed avocado, which contains high levels of MIR156a, MIR159a, and MIR169a. Notably, the concentration of miRNA ingested by avocado-fed animals was comparable to the MIR156a and MIR168a intake of rice-fed animals in the Zhang group study. Their findings confirmed the presence of miR-16, but revealed barely detectable levels of MIR156a in plasma and organ tissues, while MIR159a and MIR169a remained wholly undetectable [16].

In addition, Snow *et al.* utilized a miR-21-null (miR-21-/-) mouse strain to eloquently examine miR-21 uptake from a custom lard diet to determine whether diet-derived, endogenous mammalian miRNAs could be absorbed by the animals at any appreciable level. After 4 weeks on the lard diet, plasma and tissue RNAs were isolated from the animals and analyzed for miR-21 levels. While miR-16 was consistently detectable in plasma, miR-21 was not detectable in plasma and was present in negligible levels in organ tissues. The authors concluded that regardless of diet preparation or miRNA identity, these healthy mice were unable to maintain a steady-state level of diet-derived miRNAs in blood and body tissues [16].

### 4.4. Nonhuman Primates Failed to Absorb Plant miRNAs

Additional evidence negating the reports of diet-derived miRNA absorption was published in a report by Witwer *et al.*, in which the authors measured plant miRNA uptake in nonhuman primates. Pigtail macaques were gavage fed a soy and fruit-based protein “shake” containing an abundance of plant miRNAs and no animal-derived products. Blood samples were taken pre-gavage and at 1, 4, and 12 h post-gavage, which encompasses the timeframe of 3–6 h reported in the Zhang group study as sufficient for miRNA uptake from rice. Despite the use of novel biofluids-specific RNA purification kits and qRT-PCR, plant-specific miRNAs MIR160, MIR166, MIR167, MIR168, and MIR172 were undetectable in animal tissues. Droplet digital PCR was utilized to investigate the possible presence of extremely low levels of plant miRNAs in animal blood plasma. Detection by this method was unreliable, but revealed the potential for non-specific amplification of plant miRNAs in animal plasma [17].

In summary, multiple studies in humans, primates, mice, and insects have failed to confirm dietary miRNA uptake. The consensus of the data suggested negligible to non-detectable levels of various food-derived plant miRNAs by several highly-sensitive detection methods. This raises the concern that the results reported in the Zhang group study could reflect a lab-specific technical or experimental condition, instead of a general biological phenomenon.

## 5. Evidence Supporting Dietary Uptake of microRNAs

### 5.1. An Independent NGS Study Suggests General Presence of Foregin sRNAs from a Wide Range of Organisms in Human Bloodstream

Soon after the publication of Zhang group study, Wang *et al.* reported a NGS analysis on the small RNA (sRNA) profiles of human plasma, independently addressing whether the human circulatory system could harbor foreign miRNAs [25]. Specifically, they selected plasma samples from healthy humans, and humans with either colorectal cancer or ulcerative colitis. Their experimental design enabled them to eliminate the possibility of sample contamination to insure the correct and reliable mapping of sequencing reads. Their protocol allowed for the detection of a spectrum of sRNAs ranging from 20 to 100 base pairs. Surprisingly, their results revealed that approximately 12% of the sequences in human plasma samples originated from various exogenous species, including bacteria and fungi found in human intestinal flora, house insects, and also common plant food sources such as cereals, beans, tomato, and grapes [25].

Among the plant-derived RNAs, the most abundant sequences are from corn (Zea mays) followed by rice (Oryza sativa Japonica Group), with the number of mapped reads to corn 66 times higher on average than those to rice. However, when compared to the publicly available deep sequencing data from the serum of a Chinese individual, the relative sequence abundance between corn and rice is reversed, with the number of mapped reads to rice about 55-fold times higher than those to corn [25]. This correlates with the dietary bias of cereal *versus* rice represented in Western populations, indirectly supporting a hypothesis that these plant sRNAs are dietarily obtained.

Notably, however, the number of plant miRNA reads is significantly lower than that reported in the Zhang group study, whose data show the total number of plant miRNA reads account on average for more than 1% of total mammalian miRNA reads. Particularly, MIR168a is generally present at more than 5000 reads per million mammalian reads. Wang *et al.* estimated the total average number of reads of MIR168a to be 10 reads per 50,000 (or 200 per million) mapped human miRNA reads [25]. Thus MIR168a was detected at a level 25-fold higher by the Zhang group. This difference could be explained by variations in sequencing library preparation protocols used between the two labs. Principally, the Zhang group sequenced RNA isolated from 50 mL of human sera by an ethanol precipitation-based protocol, compared with 200 µL of sera prepared by Wang et al via a column-based protocol. Moreover, the Zhang group’s library was based on enriched miRNA fractions, while Wang *et al.* analyzed RNA samples with a broader sequence length spectrum ranging from 20 to 100 bases. Considering that the sequencing depth from the two studies was similar, and assuming equal detection efficiency of all RNA sequences in the library, the detection of a lower percentage of plant miRNAs by Wang *et al.* could be explained by the presence, and thus the diluting effect, of longer sRNAs in their library.

Interestingly, both groups observed high variation among the number of reads from individual donors for specific miRNAs. This suggests the presence of unknown factors causing variations in pathological or physiological conditions of the consumers, affecting the uptake and maintenance dynamics of exogenous RNAs, or other unknown technical reasons that introduce high variability in detection. These unidentified factors could contribute to the conflicting results obtained from miRNA feeding experiments in different labs. Furthermore, Wang *et al.* demonstrated circulating RNAs as binding to proteins or lipid structures, as proteases and detergents greatly affect their stability. This supports results presented by the Zhang group and provides a mechanistic basis to explain how circulating miRNAs could survive degradation by resident nucleases in the blood.

### 5.2. Plant miRNAs Are Found in Mammalian Milk Exosomes

Another NGS analysis of existing public milk exosomal miRNA datasets by Lukasik and Zielenkiewicz revealed the presence of plant miRNAs in animal breast milk [26]. Using porcine and human milk samples, they identified 35 and 17 plant-specific miRNA species belonging to 25 and 11 miRNA families, respectively. In the human samples, the most abundant plant-specific miRNAs were MIR166a, MIR951, MIR472a and MIR168a, while the porcine breast milk exosomes contained the highest amounts of the MIR168a, MIR156a and MIR166a.

Lukasik and Zielenkiewicz’s analysis showed that the abundance of the plant miRNAs identified were in the single digits per million total reads, which is congruent with results from Monsanto and the miRagen group [10]. There also exists a substantial amount of variation in the detection of plant miRNA across samples. For example, plant miRNA species were only identified in two out of four human breast milk sRNA libraries. This raises the concern whether the source of the plant miRNAs is cross-contamination in deep sequencing platforms. The authors propose that the disparity in plant miRNA levels between samples could argue in support of food origin of the plant miRNAs rather than cross-contamination, as the variation could potentially be attributed to donor diets. Nevertheless, the authors interpreted the presence of low-level plant miRNAs in formerly untested biofluids as potentially biologically relevant.

### 5.3. Cabbage MIR172 is Absorbed in Mice

Recently, Liang *et al.* published their findings from mouse feeding studies using cabbage (Brassica oleracea)-derived RNAs [27]. Their studies were done in parallel with the Zhang group studies and focused on MIR172, the most highly-enriched plant miRNA in cabbage. Their results showed that the precursor of MIR172 (pre-MIR172) was less stable than the mature miRNA. Mature MIR172 persisted throughout the gastrointestinal tract (stomach, intestine, fecal matter) for as long as 72 h after feeding, while pre-MIR172 was not detectable after 2 h. Furthermore, they showed that MIR172 was detected in blood and in various organs such as spleen, liver, and kidney within 2 h after feeding. The maximum surviving ratio of MIR172 in various organs was determined, with the stomach containing about 4.5%–0.4% (2–24 h after feeding), the intestines 2.4%–0.2% (2–36 h), blood about 1.3%–0.2% (2–72 h), and spleen about 0.38%–0.04% (2–72 h) of the total MIR172 orally administered. Based on these results, the absorption efficiency of cabbage MIR172 was much higher than that of MIR168a reported by the Zhang group.

However, similar to the Zhang group’s mouse feeding studies, the amount of RNA gavage fed to the mice by Liang *et al.* was outside the normal physiological range. They showed that a typical total RNA yield from cabbage leaves was about 300 µg per gram of tissue. Assuming the typical percentage of small RNA fraction constitutes only 0.1% of total RNA, gavage feeding 30 µg of small RNA is equivalent to feeding 100 g of plant tissue per feeding per mouse. Secondarily, there was no absolute quantification of miR172 or a description of an endogenous control for normalization of their qRT-PCR data. This could cause replication issues in the future.

### 5.4. Does Milk Do a miRNA Good?

Recently, Baier *et al.* tested whether mammalian milk can serve as an effective vehicle to deliver dietary animal miRNAs since miRNAs in milk are encapsulated in exosomes and are predicted to be more recalcitrant to degradation and receptive for absorption [11]. In their study, orally administered bovine milk was given to healthy human volunteers and an apparent dose-dependent response was seen for milk-based miRNAs (miR-29b and miR-200c), while no change was noted in an endogenous miRNA not found in milk (miR-1). Mice fed diets sufficient in milk miRNAs contained 61% more plasma miR-29b, than mice fed a diet depleted of milk miRNAs. Because the content of the nutrients other than miRNAs was identical in both diets, the 61% decrease in plasma miR-29b in the depleted group was explained as an insufficient supply of exogenous dietary miRNAs. The compelling message from this milk feeding study is that dietary milk-based microsomes appear to provide a mechanism for oral delivery into healthy consumers [11].

It has also been proposed that a mother’s breast milk can act as a cache of novel miRNAs for a young child, and these endogenous miRNA profiles in the milk can be altered by the mother’s dietary intake [28]. Could breastfed newborns, with their liquid diets, have an enhanced uptake capacity for dietary miRNAs?

Notably, however, the initial milk microsome experiment does have some ambiguities. For instance, the identity of most cow and human miRNAs complicates assignment of origin [29]. The endogenous level of miR-29b is high in both cow and human. Could these differences be due to confounding factors altering the regulation of endogenous miR-29b levels? In future studies, using specific genetic knockout mice deficient in a particular miRNA, in similar feeding regimes, or modifying exogenous animal miRNAs with plant-specific methylation could circumvent this ambiguity [29].

### 5.5. Dietary and Pharmacological Influences on Dietary RNA Uptake

A separate study published recently by our group demonstrated that certain dietary and pharmacological regimes appear to facilitate the detection of plant based diet-derived sRNAs in consuming animals [14]. After 3 to 7 days on a honeysuckle (*Lonicera japonica*)-containing diet, a honeysuckle-derived small RNA, MIR2911, could be detected without gavage feeding. In the honeysuckle-fed animals, MIR2911 appeared in circulation within 3 days of consumption and was no longer detecTable 48 h after the honeysuckle was removed from the diet. In the honeysuckle fed population, gavage-fed exogenous plant miRNAs could also be detected in the urine of animals. Additionally, a chemotherapeutic drug facilitated uptake of miRNAs independent of the consuming animal’s dietary history. Microscopic investigation of the intestinal villi showed that the drug, but not the honeysuckle feeding, disrupted small intestine epithelial cell organization [14]. This study proposed that certain diets or gastrointestinal injuries favor the delivery of dietary-based nucleic acids in consuming populations. However, this work does not address mechanisms of uptake and fails to address the central issue of functionality.

### 5.6. Anticancer Potential for Plant-Based miRNAs

The potential for dietary miRNAs to be chemopreventive was recently demonstrated in a miRNA feeding study [13]. Using a colon cancer mouse model, animals were given either water, an array of plant based miRNAs, or a cocktail of 3 tumor suppressor miRNAs mixed with purified plant RNAs. The suppressor miRNAs were synthesized with methyl groups on the 2’ position of the ribose of the 3’ nucleotide to mimic miRNAs of plant origin. The animals were gavaged for 28 days with 400 pmol of each tumor suppressor miRNA in the cocktail and tumor burden was subsequently measured. In the tumor suppressor-fed group, 6 of 7 mice had tumor values lower than the lowest water control fed animals. Interestingly, however, the presence of the plant mimic miRNAs in the colon could only be verified for one of the miRNAs from the cocktail [13].

These dietary miRNAs may trigger a general immune response that minimizes tumor burden independent of miRNA functionality; however, one would envision this immune response would be similar in the animals fed the plant-based miRNAs or the tumor suppressor cocktail. Interestingly, there was only a slight tumor reduction feeding the non-specific plant RNAs compared to the water controls, suggesting that the tumor suppressing capability of the gavage fed tumor suppressor miRNAs were sequence specific.

Certainly a knee-jerk response to these studies is “increase the sample size”; however, despite this obvious limitation, this study is groundbreaking in that it alters our concept of the relationship between health and nutrition and potentially opens up new vistas for gene therapy.

### 5.7. Plant Small RNAs as Antivirals

The most elaborate evidence of the efficacy of miRNA in therapeutics comes from a recent study out of Zhang group, the same lab who published the initial study on cross-kingdom dietary gene regulation by plant dietary miRNAs [12]. When animals were fed boiled honeysuckle decoction, the MIR2911 from the decoction seems to be stable enough to survive the preparation process, withstand digestion and be delivered to the circulation and lungs of the consuming animals. Furthermore, this plant-based small RNA is biologically active *in vivo* as the nucleic acid suppresses influenza A viruses by inhibiting the replication process.

One may envision that boiling honeysuckle would degrade MIR2911; however, this particular small RNA appears resilient to degradation [12]. This resiliency and the biological effects have lead this group to suggest MIR2911 maybe a ‘virological penicillin’ to treat numerous viruses.

In summary, various studies, including the bovine milk, honeysuckle and tumor suppressors’ studies all suggest that certain conditions may favor delivery and functionality of dietary miRNAs.

## 6. Implication of Dietary microRNAs Regulating Consumer Genes

Plants, like animals, express an abundance of miRNAs in all tissues, including edible portions (See Table 1 for the top expressed dietary miRNAs from various crops, fruits, and vegetables). Assuming the absorbed plant miRNAs behave like animal endogenous miRNAs, where only partial sequence complementarity is needed for effecting target gene regulation, the impact of dietary miRNA on animal metabolism and development could be substantial. It is attractive to speculate that eating specific plant-based foods could be a novel, economic, and “natural” means to deliver miRNA disease suppressors to target tissues, or to fine-tune human health as atypical nutrients. If this scenario develops, the current data suggest that the kinetics of absorption will need to be greatly optimized and delivery to specific tissues enhanced. We favor a model where dietary regimes and physiological conditions that enhance absorption and limit excretion could support the functionality of dietary-based nucleic acids in consuming populations. Establishing these conditions could drastically alter our concept of the relationship between health and nutrition, help establish useful dietary practices, and potentially open up new vistas for gene therapy.

**Table 1:** Highly-expressed dietary miRNAs from various crops, fruits, vegetables.

Species	Highly Expressed microRNAs
Rice (*Oryza Sativa*)	osa-miR156a, osa-miR168a, osa-miR1846e, osa-miR167d, osa-miR168b [30]
Corn (*Zea May*)	zma-miR156a, zma-miR168a, zma-miR 169c, zma-miR399e, zma-miR167a, zma-miR156 [31]
Soybean (*Glycine max*)	gma-MIR3522b, gma-MIR-1507a, gma-MIR 1509a, gma-MIR482*, gma-MIR-1510a-5p, gma-MIR167d, gma-MIR 166a, gma-MIR-166b; gma-MIR396e [32]
Tomato (*Solanum lycopersicum*)	miR159 a, miR162b, miR168a, miR164c, miR164d [33]
Barley (*Hordeum vulgare*)	hvu-miR168, hvu-miR156, hvu-miR167, hvu-miR165/166, hvu-miR172, hvu-miR2005 [34]
Wild wheat (*Triticum dicoccoides*)	miR159, miR1450, miR319, miR896, miR168, miR474, miR167, miR164, miR528, miR1436, miR396 and miR894 [35]
Peanut (*Arachis hypogaea*)	ahy-miR157a, ahy-miR156a, ahy-miR168a, ahy-miR166a, ahy-miR166h, ahy-miR167f [36]
Sweet Orange (*Citrus sinensis*)	csi-miR168a, csi-miR172a, csi-miR166j, csi-miR167a, csi-miR157a, csi-miR479, csi-miR156a [37]
Oilseed (*Brassica napus*)	miR156, miR157, miR168a, miR166, miR167, miR399 [38]

Agrobiotechnologists are constantly engineering crops to express high levels of novel small RNAs to control plant diseases and increase crop yield [39]. The current scientific dogma proposes that consumption of dietary RNAs has no influence on health [24]. However, evidence is emerging that miRNAs can function in non-canonical manners to suppress gene expression [40,41]. While one could be tempted to speculate that low copy number miRNAs could exert regulatory influence, biological effects are difficult to imagine [22]. As Snow *et al.* observed, dietary plant miRNAs, even if reliably quantifiable, are present at fewer than one copy per cell in healthy target organs [16]. While we can speculate about the repertoire of miRNA-mediated regulation, currently the field does not have the assays available to firmly establish regulatory effects.

On the other hand, if dietary miRNA regulation of consumer gene regulation is established, we must also evaluate potential risks in the new RNA technologies. The risks experts identify are not usually the risks that upset the public [42]. Public fears and perceived risk increase if the issue seems to be poorly understood by science and is subject to contradictory statements from responsible sources [43,44,45]. This is certainly the case with genetically modified (GM) crops in general. Continuous and ardent opposition to GM foods has had serious repercussions for plant research, for the commercial development of new crop varieties and, most importantly, for developing countries that could benefit most from this technology. Food safety advocates are constantly asking the U.S. Department of Agriculture to slow the approval processes of GM crops. Research to date has certainly provided conflicting results on the extent and importance of dietary RNA influence in mammals. Certainly, restricted expression of sRNAs will reduce the risk of exposure via dietary intake. For example, it is easy to envision a scenario whereby agbiotechnology companies limit expression of novel RNA to non-edible tissues to drastically reduce the risk of dietary exposure. However, given the current trend in agbiotechnology, such precautions will only be implemented after research has convincingly documented consumer uptake.

## 7. Future Directions

**Figure 1:**
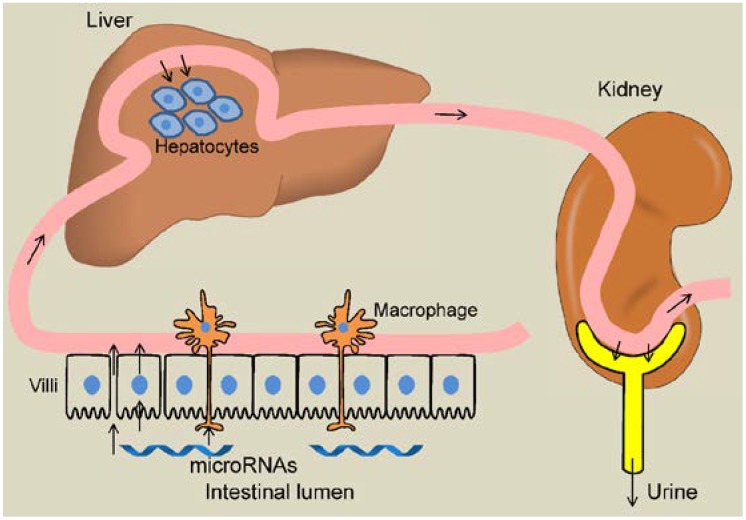
Model of dietary microRNA dynamics within the consumer. Dietary microRNAs, in order to be detected in circulation and tissues, must circumvent degradation by the gut; transverse the intestinal barrier (possibly through active transport across intestinal epithelial layers, via leaky gut lining, or trafficked by the immune cells patrolling the gut); withstand degradation in circulation; survive sequestration into and metabolism by the tissues such as the liver; and survive filtration and excretion at the kidney.

For any nutrient, the circulation or tissue level depends on the following parameters: food processing and intake, degradation and processing by the GI tract, uptake and transport across the intestinal and the ensuing tissue barriers, stability in circulation, sequestration and metabolism by tissues such as liver, and excretion by kidney. It is possible under certain pathological conditions, the consumer’s gut can be modified in a way to promote stability of dietary miRNAs in the GI tract and enhance the absorption into the blood stream. These conditions could include suppressed digestive enzymes, increased permeability of the gut lining, decreased degradation by the liver and decreased excretion by the kidneys (see Figure 1 for our model of uptake and dynamics of plant dietary microRNAs in mammals).

Since the main concerns for dietary miRNA studies arise from high variation and low copy number detection, more judicious detection techniques need to be developed with more reliable normalizing sequences for quantification of exogenous miRNAs. Development of extremely sensitive tissue sensors (as used in *C. elegans* [32]) could help identify both the presence and functionality of low-level absorbed miRNAs. In addition, the molecular component involved in miRNA transport needs to be examined in animals.

## 8. Conclusions

Over two years have passed since the original publication demonstrating the ability of dietary miRNAs to transverse the mammalian gut and regulate an animal gene in the liver [8]. While several studies have suggested this is a possible mode of cross-kingdom gene regulation, the majority of work has questioned the validity of this report [10,16,46]. The majority of the data suggests that gastrointestinal uptake of dietary plant miRNA is not occurring in healthy consumers and the measured tissue and blood dietary miRNA levels that have been reported are so limited that their dietary impact are negligible. However, recent studies [12,13,14] appear to be establishing conditions that allow miRNAs to overcome the barriers for entry into consumers (See Table 2 for a summary of evidence for and against the hypothesis of dietary miRNA uptake and function in mammals). With the number of people worldwide living under pathological or adverse nutritional conditions, such research remains a valid endeavor considering the potentially significant impact of absorbed miRNAs on gene expression, and implications for clinical application, nutrition, and agriculture.

**Table 2:** Summary of evidence regarding dietary microRNA uptake and functionality in consumers.

Dietary microRNA Uptake and Function in Mammalian Consumers?	
Evidences Against	Exogenous levels in serum are inconsistent and typically low. Various feeding studies from different labs failed to show absorption of dietary microRNAs. Target suppression is shown only in the initial study, and was not replicated by another group. In silico analyses suggest plant microRNA reads in animal tissue could be due to contamination.
Evidences For	Oral uptake of exogenous microRNAs is well-characterized in nematodes and insects (indirect evidence). Detection of fungal, bacterial, and plant derived microRNAs in mammalian circulation is consistently reported. MIR168a target suppression shown in the initial study. Detection of MIR172 in circulation and various organs in mice fed cabbages. Report of Bovine milk microsome-derived microRNAs being absorbed in humans and mice. Detection of honeysuckle derived small RNA MIR2911 in mouse circulation and urine, and report of antiviral functions of MIR2911 in vivo in mice. Report of tumor suppressing effect from orally fed synthetic tumor suppressor microRNAs with plant microRNA chemistry.
